# Delayed Post-Anoxic White Matter Injury in an Infant

**DOI:** 10.5334/jbsr.2126

**Published:** 2020-06-22

**Authors:** Bert Degrieck, Julie Dutoit, Nele Herregods

**Affiliations:** 1 Ghent University Hosptial, BE

**Keywords:** postanoxic leukoencephalopathy, delayed white matter injury, child, hypoxic brain injury, Magnetic Resonance Imaging (MRI)

## Abstract

**Teaching Point:** White matter reversal on diffusion-weighted magnetic resonance imaging is indicative of delayed post-anoxic encephalopathy. This manifests sooner in young infants than in adults.

## Case report

**Figure 1:**
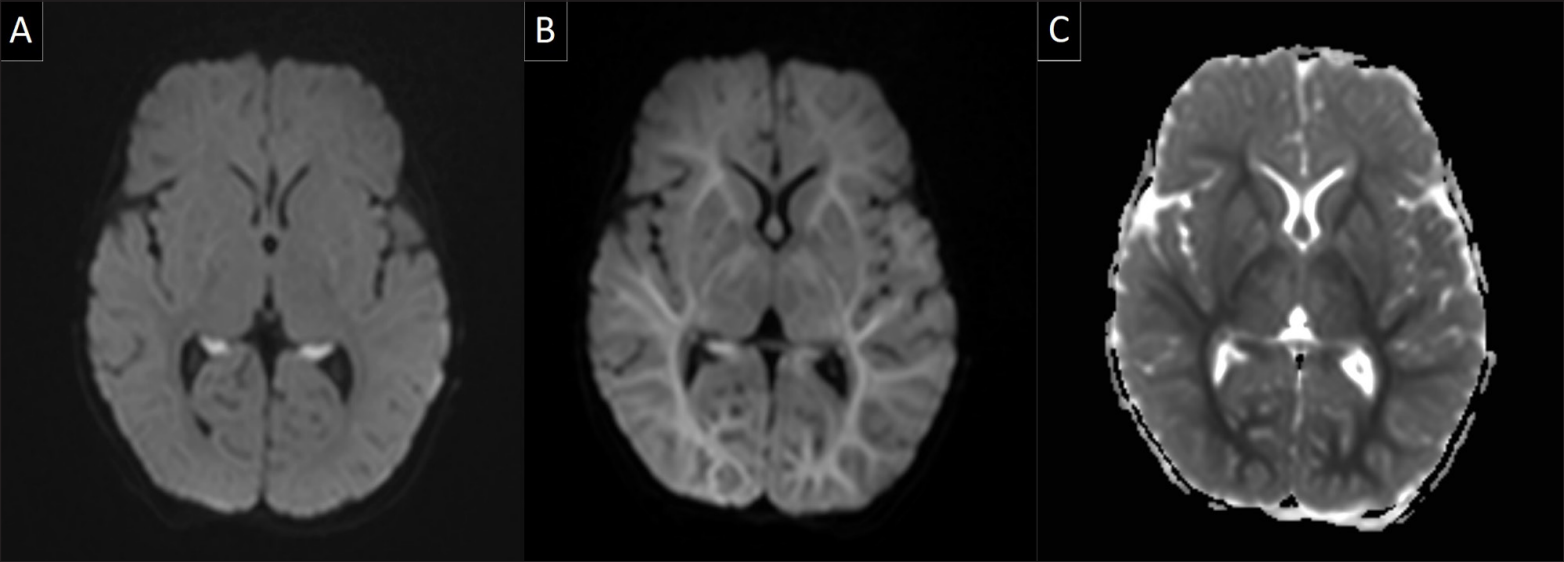
**A.** Axial diffusion weighted MR image (B1000) of the brain 1 day after the hypoxic-ischaemic event occurred shows extensive restricted diffusion in both hippocampi. There is no white matter involvement visible. **B.** Axial diffusion weighted MR image (B1000) of the brain 3 days after the hypoxic-ischaemic event occurred shows delayed post anoxic white matter injury with the white matter reversal sign: there is new extensive and symmetric diffuse high signal in the superficial and deep white matter, with relative sparing of the frontal white matter. **C.** Axial ADC image shows low ADC values in the affected white matter, confirming the restricted diffusion.

A four-month-old male infant suffered from near drowning and was resuscitated by a parent for five minutes before medical assistance arrived. An intraosseous line was placed in the right tibia and the patient was immediately transported to the hospital. During transport, resuscitation was continued for 15 minutes before a return of spontaneous circulation was established, and in this period, there were four administrations of 0.6cc of adrenaline 1/10000. Upon arrival at the emergency department, the pupils were dilated and not reactive to light. The patient was sedated, intubated, and transported to the paediatric intensive care unit. Progressive hypertension was observed in the hours following admittance, and cytotoxic cerebral oedema was suspected. Brain magnetic resonance imaging (MRI) was performed the next day, showing restricted diffusion in both hippocampi and to a lesser extent both globi pallidi (Figure 1A, diffusion-weighted imaging). There was no involvement of the white matter, and no other abnormalities were visible. The next days of hospitalization the situation worsened, with absence of reflexes and no reaction to pain stimuli after cessation of sedation. Electroencephalogram (EEG) showed cortical activity but no convulsive activity. Because of tachycardia and tachypnoea, morphine was administered. To further determine the patient management, brain MRI was repeated on the third day of admittance (Figure 1B, diffusion-weighted imaging and Figure 1C apparent diffusion coefficient map). It showed new, extensive, and symmetric restricted diffusion in the supratentorial deep and subcortical white matter, indicating delayed post-anoxic encephalopathy with extensive cytotoxic white matter edema. Additionally, there was a new focus of restricted diffusion in the splenium of the corpus callosum and the known diffusion restriction in the hippocampi and globi pallidi also persisted. Repeat EEG the same day only showed a flat monorhythmic curve and no convulsive activity. Because of poor prognosis, palliative sedation was started and the patient passed away the next day.

## Discussion

Diffusion abnormalities are the earliest visible signs of hypoxic ischemic brain injury on MRI, visible as soon as one hour after the event. Post-anoxic leukoencephalopathy or delayed white matter injury is an uncommon syndrome that manifests in the subacute stage or even later, without any white matter changes visible on MRI in the acute phase (the first hours following the event) [1]. This delayed white matter reversal on DWI, with white matter having a higher diffusion restriction than the grey matter after the acute stage has passed, is therefore indicative of delayed post-anoxic leukoencephalopathy and can be seen in all age groups. In young children this pattern of white matter changes can occur as early as two days after the event. In adults this is seen later, in the weeks following the hypoxic injury. The underlying mechanism for delayed white matter injury is not completely understood, and the pathophysiology might be similar to Wallerian degeneration. The majority of patients recover at least partially. Additional involvement of the deep grey matter results in a poorer outcome. Diffusely atrophic changes can be seen on follow-up imaging in surviving patients.

## References

[1] Huang BY, Castillo M. Hypoxic-ischemic brain injury: Imaging findings from birth to adulthood. Radiographics. 2008 Mar-Apr; 28(2): 417–439. DOI: 10.1148/rg.282075066 18349449

